# Oxidative stress and endoplasmic reticulum (ER) stress in the development of neonatal hypoxic–ischaemic brain injury

**DOI:** 10.1042/BST20170017

**Published:** 2017-09-22

**Authors:** Claire Thornton, Ana A. Baburamani, Anton Kichev, Henrik Hagberg

**Affiliations:** 1 Perinatal Brain Injury Group, Centre for the Developing Brain, Division of Imaging Sciences and Biomedical Engineering, King's College London, King's Health Partners, St. Thomas’ Hospital, London SE1 7EH, U.K.; 2 Perinatal Center, Institute of for Clinical Sciences and Physiology and Neurosciences, Sahlgrenska Academy, University of Gothenburg, Gothenburg 41685, Sweden

**Keywords:** endoplasmic reticulum stress, hypoxic–ischaemic brain injury, neonatal, reactive nitrogen species

## Abstract

Birth asphyxia in term neonates affects 1–2/1000 live births and results in the development of hypoxic–ischaemic encephalopathy with devastating life-long consequences. The majority of neuronal cell death occurs with a delay, providing the potential of a treatment window within which to act. Currently, treatment options are limited to therapeutic hypothermia which is not universally successful. To identify new interventions, we need to understand the molecular mechanisms underlying the injury. Here, we provide an overview of the contribution of both oxidative stress and endoplasmic reticulum stress in the development of neonatal brain injury and identify current preclinical therapeutic strategies.

## Introduction

Term neonates (>37 weeks gestation) who develop hypoxic–ischaemic encephalopathy (HIE), neonatal encephalopathy or perinatal arterial stroke have a greater incidence of cerebral palsy, cognitive impairment, developmental delay and epilepsy [1–3]. Clinical MRI studies report that perinatal hypoxic–ischaemic brain injury is characterised by lesions in grey matter structures such as the basal ganglia, thalamus and cortex, and to a lesser extent infarctions in white matter, where increasing severity is predictive of poorer neurodevelopmental and motor outcome [4–6]. Currently, the only available treatment is therapeutic hypothermia, where if initiated within 6 h can improve neurological outcome [4,7]. The underlying mechanisms contributing to the development of perinatal brain injury have not been entirely elucidated.

Studies from small and large animal models have shown that with advancing gestation, the developing brain is increasingly vulnerable to hypoxia–ischaemia (HI), with term age-equivalent models showing greater vulnerability to neuronal cell death [8,9] in part due to their high metabolic demand [9–11]. Following HI, cells initially undergo impaired cerebral oxidative metabolism, swelling and extracellular accumulation of excitatory amino acids (primary phase) and transiently recover (latent phase) prior to a period of secondary energy failure (secondary phase). Over subsequent hours to days, the injurious mechanisms of the secondary phase lead to neuronal cell death. These include excitotoxicity, deterioration of mitochondrial function, inflammation, increased reactive oxygen species (ROS) production and activation of nitric oxide synthase (NOS) and accumulation of intracellular Ca^2+^ [12–15]. We and others have identified mitochondrial dysfunction as the intracellular hub of these injury responses following HI in the immature brain [16].

Mitochondria are key determinants of cell fate having the ability to induce cell death. Delayed cell death that follows neonatal HI is morphologically characterised by either apoptotic (programmed), necrotic (uncontrolled) and more recently, necroptotic (programmed necrosis) phenotype [11,16–21]. In comparison with the adult, the immature brain has a high expression of many pro-apoptotic proteins (including Bax, caspase-2, -3, -8 and -9) [22,23], and apoptosis occurs following mitochondrial outer membrane permeabilisation (MOMP). Briefly, Bax translocates from the cytosol to form Bax–Bak pores at the outer membrane which allows for the release of pro-apoptotic proteins. Cytochrome *c*, apoptosis-inducing factor (AIF), smac/Diablo and endonuclease G translocate from the mitochondria to the cytosol, and while each has a different downstream target, all ultimately lead to apoptotic cell death [20–22,24–26]. Necroptosis is linked with apoptosis as ligands such as tumour necrosis factor-alpha, TNF-related apoptosis-inducing ligand and Fas can activate death receptors in both instances. However, caspase-8 appears to be the checkpoint that determines whether a cell undergoes necroptosis (caspase-8-independent) or apoptosis [20,27]. Cell death is obviously the endpoint of the induction of these pathways, but in order to design intelligent intervention, a clear understanding of the mechanisms leading to the triggering of such signalling is required. Oxidative stress and endoplasmic reticulum (ER) stress responses are rapidly induced following HI and contribute to cellular injury and death in the immature brain. Here, we will briefly outline how these stresses are generated in the developing brain after HI and the potential for therapeutic intervention.

## Oxidative stress and HIE

**Figure 1.**
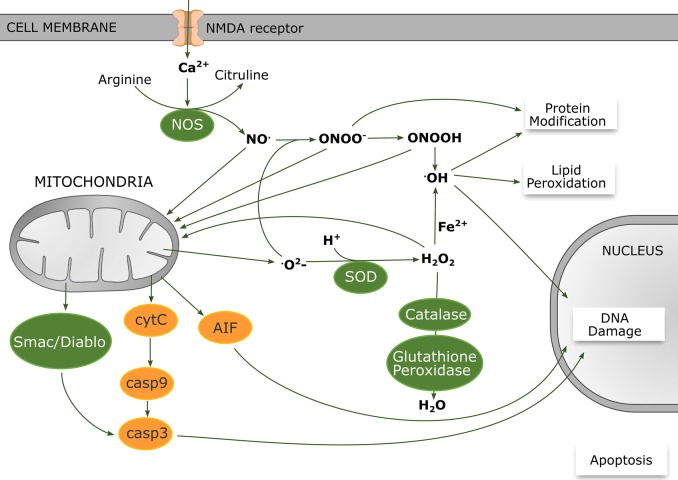
Schematic diagram of the production of ROS and nitrogen species following HI. Increased intracellular calcium active NOS to generate NO. Superoxide ($O_{2}{}^{\cdot -}$) interacts with NO to form ONOO^−^ and subsequently leads to generation of ^·^OH^−^. ONOO^−^ itself can target the mitochondrial respiratory complexes directly. SOD also converts $O_{2}{}^{\cdot -}$ into H_2_O_2_, where excessive build-up of H_2_O_2_ leads to an exacerbated generation of ^·^OH^−^ via the Fenton reaction. All of these reactive oxygen and nitrogen species contribute to lipid peroxidation, DNA damage and harmful protein oxidation in addition to influencing the release of pro-apoptotic proteins from the mitochondria to initiate cell death.

Oxidative stress occurs when the generation of ROS and reactive nitrogen species (RNS) exceeds the capability of endogenous antioxidant systems. Under normal physiological conditions, low concentrations of ROS and RNS can act as signalling molecules [28]. However, during reperfusion following the primary phase of HI, there is a rapid production of free radicals. Aberrant glutamate receptor activation results in calcium influx activating neuronal nitric oxide synthase (nNOS) to generate nitric oxide (NO; Figure 1). Superoxide ($O_{2}{}^{\cdot -}$), formed from leakage of electrons from complexes I and III of the electron transport chain, interacts with NO to form peroxynitrite (ONOO^−^), leading to generation of hydroxyl radicals (^·^OH^−^); subsequent ^·^OH^−^-mediated cellular impairment occurs through lipid peroxidation, protein oxidation, DNA damage and inhibition of mitochondrial function (specifically complex IV). Complexes I and IV are also targets for direct damage by ONOO^−^ (Figure 1). Excitotoxicity-mediated calcium influx also triggers the formation of $O_{2}{}^{\cdot -}$ through the action of NADPH oxidase (NOX, [29]). $O_{2}{}^{\cdot -}$ can be neutralised by superoxide dismutase (SOD), which converts $O_{2}{}^{\cdot -}$ into hydrogen peroxide (H_2_O_2_), subsequently converted into H_2_O by catalase. However, excessive build-up of H_2_O_2_ can lead to further generation of ^·^OH^−^ through the iron-based Fenton reaction.

The immature brain has a high oxygen consumption and consists of a high concentration of free iron, water content and easily oxidised unsaturated fatty acids. In addition, low myelination and a low expression of antioxidant enzymes [SOD and glutathione peroxidase (GPx)] consequently lead to an underdeveloped antioxidant system, rendering it particularly vulnerable to oxidative damage, with both ROS and RNS strongly influencing excitotoxicity, cell death and mitochondrial impairment [30–34].

Studies of neonates with HIE identified evidence of oxidative stress [35,36] as well as evidence suggesting that therapeutic hypothermia reduced lipid peroxidation among its many benefits [36]. It is therefore critical that mechanisms specific to immature brain are identified in order to develop interventions. NOS and mitochondrial electron leakage are believed to be the major contributors of ROS/RNS in the immature brain [37,38] Following HI in the neonatal rat, there is an increase in expression of hydroxyl radicals and free iron in ischaemic regions, and it was recently established that this was mediated in part by nitric oxide [39,40]. Both endothelial nitric oxide synthase and nNOS are significantly increased in the hours following HI [41,42]. Interestingly, the developmental expression pattern of nNOS is comparable to regions vulnerable to injury, and nNOS knockout mice show less injury in response to HI [43–46]. Modulating expression of H_2_O_2_ has further implicated ROS as detrimental following neonatal HI. Detoxifying enzyme GPx1, which decreases H_2_O_2_ levels, has been shown to be protective to neonatal brain after HI [47], whereas overexpression of H_2_O_2_ which occurs in mice that overexpress Cu/Zn SOD1, required to produce H_2_O_2_, is associated with exacerbation of the injury after HI [48]. This could be due to the accumulation of H_2_O_2_ being exposed to high levels of free iron in the immature brain moving the Fenton reaction towards generation of hydroxyl radicals (Figure 1). The role of NOX is less clear in the immature brain. Superoxide production is rapidly increased in the immature mouse brain after HI, coinciding with an increase in the expression of NOX subunits. This increase is functionally coupled to phosphorylation of the NMDA receptor 2B (NR2B) subunit as mutation of this NR2B phosphorylation site reduces HI-induced up-regulation of expression of NOX2, suppresses superoxide formation and reduces cell death [29]. However, genetic ablation of NOX subunits directly has no effect on infarct size and may exacerbate brain damage in excitotoxicity models of immature injury [49]. Clearly, the complexity of these signalling pathways coupled with contributions from different neural cell types makes it difficult to determine the extent on NOX involvement.

Mitochondria are a major contributor to the generation of ROS but also sensitive to oxidative stress, which can culminate in cell death (Figure 1) [38,50]. The release of cytochrome *c* from the mitochondria is a crucial step in induction of the apoptotic pathway. For this to occur, it must be dissociated from the inner mitochondrial membrane where it is bound to cardiolipin (rich in polyunsaturated fatty acids). Numerous studies suggest that increased ROS generation promotes cytochrome *c* release from the inner mitochondrial membrane by acting as a catalyst in the oxidation of cardiolipin [51–53]. Independent of this, NO, peroxynitrite and 4-hydroxynononeal, a product of lipid peroxidation, have been shown to enhance mitochondrial permeabilisation [54]. Downstream, once initiated, both caspase activation and necroptosis have been suggested to exacerbate production of ROS [55,56].

Given that induction of ROS and RNS is an early event in the development of injury, it is unsurprising that antioxidant therapies are being explored to combat neonatal HI-mediated brain injury. Among its many effects, melatonin acts to scavenge free radicals and to induce expression of antioxidants. Its neuroprotective action was promising in both small and large animal models of neonatal HI [57,58], and this has led to the initiation of clinical trials. However, translation from immature preclinical models to clinical trials is not always straightforward as a mixed cell population such as the brain can lead to differential responses. This was highlighted recently in trials of erythropoietin (EPO), where previous studies showed a high degree of neuroprotection in neonatal models of HI hypothesised to be due to suppression of oxidative stress [59,60]. Very recent studies identified that excessively high H_2_O_2_ expression resulted in EPO treatment worsening injury [61], and that differential effects were observed in astrocytes compared with microglia [62]. In a similar manner, allopurinol, a free radical scavenger, provided beneficial effects in animal models [63], which were difficult to reproduce consistently in clinical trials (reviewed in ref. [64]). Other antioxidants under consideration as neuroprotectants in immature brain after HI include the plant polyphenol resveratrol [65] and *n*-acetylcysteine [66–69].

## ER stress and HIE

**Figure 2.**
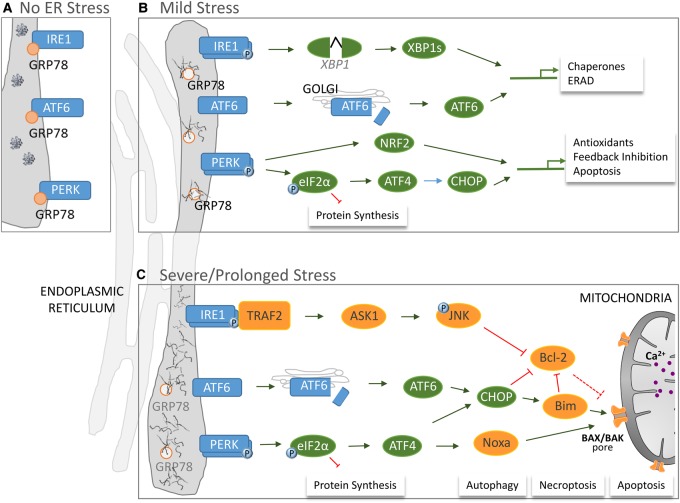
The response of the ER during homeostasis and under mild and chronic stress. (**A**) During homeostasis when normal protein folding occurs, chaperone protein glucose-related protein (GRP)78 binds transmembrane proteins IRE1, ATF6 and PERK in the ER. (**B**) Following mild stress leading to misfolding of proteins, the ER initiates the UPR cascade, releasing GRP78 to chaperone proper protein folding. Activated transmembrane proteins damp down protein synthesis except for proteins required for the UPR to restore equilibrium. (**C**) Severe, prolonged stress to the ER can induce cell death that overwhelms the UPR and culminates in cell death through many mechanisms.

The ER functions to synthesise and regulate protein folding of transmembrane and secreted proteins as well as synthesising phospholipids and cholesterol, but, in addition, it is the site of the highest intracellular calcium concentrations [70]. Therefore, it is unsurprising that pathological alterations in a wide range of intracellular mechanisms culminate in ER stress, manifested as disturbed protein folding and accumulation of unfolded proteins in the ER lumen. To maintain cellular homeostasis, the ER triggers the unfolded protein response (UPR) [71,72] mediated by the chaperone protein glucose-regulated protein (GRP)78 also known as binding immunoglobulin protein. GRP78 is localised in the lumen of the ER and is required for numerous ER functions [73]. Normally, GRP78 binds to and inhibits the activation of the UPR sensors inositol-requiring enzyme (IRE)1, protein kinase RNA-like endoplasmic reticulum kinase (PERK) and activating transcription factor (ATF)6, transmembrane proteins, which possess GFP78-binding sites in their ER lumen portions (Figure 2A). However, accumulation of unfolded proteins results in the sequestering of GRP78 away from these UPR sensors leading to regulation of protein synthesis and increased degradation of unfolded proteins, either through autophagy or ER-activated degradation (ERAD).

Once released from GRP78, IRE1 oligomerises, becomes phosphorylated and utilises its endoribonuclease domain to degrade RNA, reducing the burden on the ER. In addition, IRE1 splices and activates XBP1, which translocates to the nucleus, up-regulating expression of key proteins required to manage both the stress response (e.g. chaperones GRP78 and GRP94) and improved clearing of unfolded proteins (ERAD) [74]. In addition to degradation by ERAD, both PERK and IRE1 can trigger autophagy in response to ER stress. Oligomerised, phosphorylated PERK down-regulates general protein synthesis through phosphorylating and inactivating elongation initiation factor (eIF)2α. However, translation of selective mRNAs is up-regulated after eIF2α inhibition, e.g. ATF4, which can induce the expression of C/EBP-homologous protein (CHOP). PERK also phosphorylates NRF2 resulting in its activation and increased antioxidant response [75]. Finally, ATF6 translocates to the Golgi where it is cleaved, becoming an active transcription factor capable of up-regulating expression of chaperones, as well as CHOP and XBP1 (Figure 2B) [76].

Pathological signalling events triggered by birth asphyxia disrupt cellular homeostasis and as such lead to the induction of ER stress and the UPR [77]. Using a well-characterised rat model of neonatal HI, Puka-Sundvall et al. [19] demonstrated that there is a significant accumulation of calcium in the ER with clear increases from 30 min to 3 h following HI injury. In cortical neurones subjected to oxygen-glucose deprivation (an *in vitro* mimic of HI), phosphorylation of PERK, IRE1 and cleavage of ATF6 is observed to occur rapidly after injury [78]. Phosphorylation of PERK and eIF2α also occurs rapidly and transiently *in vivo* after neonatal HI in term-equivalent mice as well as an up-regulation of GRP78 and CHOP [78–81].

Although the aim of the UPR is to restore proteostasis and cellular homeostasis, severe ER stress and prolonged activation of the above pathways can induce cell death (Figure 2C) [76]. Prolonged activation of PERK can result in expression of Noxa which induces Bax localisation to the mitochondrial outer membrane resulting in its permeabilisation and induction of apoptosis [82]. Bax-mediated mitochondrial outer membrane permeabilisation is a hallmark of secondary brain injury in neonatal HI [23,83]. Similarly, ATF6 will induce pro-apoptotic Bim expression [84], while IRE1 recruits TRAF2 and downstream JNK signalling to inhibit anti-apoptotic Bcl-2 protein expression [85]. Induction of death receptor expression is also a consequence of prolonged ER stress (Figure 2C) [86]. As all these pro- and anti-apoptotic effects are observed in the development of injury in neonatal HI [87–90], it is tempting to speculate that targeting prolonged ER stress may represent an intervention point for the early inhibition of cell death mechanisms triggered after neonatal HI.

Although the mechanisms are not as clearly defined, prolonged ER stress can also induce the up-regulation of autophagy and phosphorylation of key signalling molecules in the necroptotic (regulated necrosis) cell death pathway. Inhibition of necroptosis is neuroprotective after HI *in vivo* [91], and ER stress is reported to function upstream of necroptosis in this model [92]. Prolonged ER stress can also result in cross-talk with autophagy when the ERAD degradation of proteins becomes overwhelmed [93]. In animal and cellular models of mitochondrial respiratory complex diseases, both ER stress and autophagy were recently found to be up-regulated as a consequence of disease-induced translation dysfunction and proteotoxic stress [94]. This elegant study by Peng et al. found that rather than targeting the complexes themselves, significant mitochondrial functional benefits were instead conferred by inhibiting the downstream consequences. In particular, low-dose cycloheximide and probucol (an anti-hyperlipidemic drug in clinical use) were very effective in preventing the induction of ER stress and the up-regulation of autophagy, promoting oxidative phosphorylation, cell survival *in vitro* and reduced disease severity *in vivo* [94]. Aberrant autophagy is also reported in the *in vivo* model of HI, although whether the pathway acts in a beneficial or deleterious manner is currently unclear [81,95,96].

Compared with the field of oxidative stress in neonatal HI (see above), there are significantly fewer neuroprotective interventions targeting ER stress. However, many recent studies have identified neuroprotective paradigms in *in vivo* rodent models of neonatal HI, which act through the prevention of prolonged ER stress (Table 1). None of these neuroprotectants targets ER stress uniquely (e.g. melatonin also act as a free radical scavenger), and it remains to be seen whether these potential therapies will offer synergistic efficacy with hypothermia, the current standard of care, or whether they represent ‘stand-alone’ treatments.

**Table 1:** Reducing ER stress is neuroprotective in rodent models of neonatal HI

Treatment	Species/delivery	Outcome	Ref.
Basic fibroblast growth factor (bFGF)	Rat (P7)/intranasal	Reduced ER stress (ATF6, GRP78, XBP-1, ATF4 and CHOP) Reduced infarct volume	[80]
Acidic fibroblast growth factor (aFGF)	Rat (P7)/intranasal	Reduced ER stress (ATF6, GRP78, XBP-1, ATF4 and CHOP) Reduced infarct volume	[100]
Necrostatin-1 (Nec-1)	Mice (P7)/intracerebroventricular	Reduced ER stress (GRP78, PERK, peIF1a, XBP1, GADD34 and CHOP) EM (organelle pathology)	[92]
Hydrogen-rich saline	Mice (P7)/intraperitoneal	Reduced ER stress (GRP78 and CHOP) Reduced infarct volume	[101]
Notoginsenoside R1 (NGR1)	Rats (P7)/intraperitoneal	Reduced ER stress (GRP78, PERK, IRE1α, CHOP and BCL-2) Reduced infarct volume	[79]
Melatonin	Rats (P7)/intraperitoneal	Reduced ER stress (GRP78, PERK, IRE1α, CHOP and XBP-1) Reduced infarct volume	[102]

## Conclusion

The molecular mechanisms underlying the development of brain injury in neonates who have suffered birth asphyxia remain unclear and providing treatment options is a critical, unmet clinical need. The recent avalanche of preclinical data has identified many key pathways activated early in the development of the pathology and which may be amenable to therapeutic intervention. Here, we have distilled the recent evidence regarding induction of oxidative stress and ER stress signalling after HI injury in the immature brain. More is currently understood about oxidative stress than ER stress in the immature brain. Mice genetically modified to alter the expression of the ER transmembrane receptors may provide more specific clues as to the contribution of ER stress in the development of neonatal brain injury. However, it is impossible to consider these areas in isolation. Although due to their complexity it is hard to evaluate this cross-talk experimentally, there are a few examples emerging. For example, induction of ER stress in macrophages is reported to result in increased NOX expression and subsequent apoptosis is mediated by CHOP [97], and a member of the GPx family promotes the function of GRP78 [98]. More generally, ROS can also be produced in the ER as a by-product of protein folding, upsetting the critical redox balance within the ER lumen, inducing downstream oxidative stress responses and disrupting cellular homeostasis which feeds back to exacerbate ER stress further [99]. Identifying drugs which can tackle multiple targets within these pathways, e.g. melatonin, may prove to be the key in the development of potential interventions to ameliorate brain injury in these vulnerable neonates.

## Abbreviations

^·^OH^−^: hydroxyl radicals
ATF: activating transcription factor
CHOP: C/EBP-homologous protein
eIF: elongation initiation factor
eNOS: endothelial nitric oxide synthase
EPO: erythropoietin
ER: endoplasmic reticulum
ERAD: ER-activated degradation
GPx: glutathione peroxidase
GRP: glucose-regulated protein
H_2_O_2_: hydrogen peroxide
HI: hypoxia–ischaemia
HIE: hypoxic–ischaemic encephalopathy
IRE: inositol-requiring enzyme
nNOS: neuronal nitric oxide synthase
NO: nitric oxide
NOS: nitric oxide synthase
NOX: NADPH oxidase
NR2B: NMDA receptor 2B
$O_{2}{}^{\cdot -}$: superoxide
ONOO^−^: peroxynitrite
PERK: protein kinase RNA-like endoplasmic reticulum kinase
RNS: reactive nitrogen species
ROS: reactive oxygen species
SOD: superoxide dismutase
UPR: unfolded protein response
